# Fretting Fatigue with Cylindrical-On-Flat Contact: Crack Nucleation, Crack Path and Fatigue Life

**DOI:** 10.3390/ma10020155

**Published:** 2017-02-10

**Authors:** Nitikorn Noraphaiphipaksa, Anchalee Manonukul, Chaosuan Kanchanomai

**Affiliations:** 1 Center of Materials Engineering and Performance, Department of Mechanical Engineering, Faculty of Engineering, Thammasat University, Pathumthani 12120, Thailand; nitikorn@engr.tu.ac.th; 2 National Metal and Materials Technology Center (MTEC), National Science and Technology Development Agency (NSTDA), 114 Thailand Science Park, Paholyothin Rd., Klong 1, Klong Luang, Pathumthani 12120, Thailand; anchalm@mtec.or.th

**Keywords:** cylindrical-on-flat contact, fretting fatigue, crack nucleation, crack closure, fatigue life

## Abstract

Fretting fatigue experiments and finite element analysis were carried out to investigate the influence of cylindrical-on-flat contact on crack nucleation, crack path and fatigue life of medium-carbon steel. The location of crack nucleation was predicted using the maximum shear stress range criterion and the maximum relative slip amplitude criterion. The prediction using the maximum relative slip amplitude criterion gave the better agreement with the experimental result, and should be used for the prediction of the location of crack nucleation. Crack openings under compressive bulk stresses were found in the fretting fatigues with flat-on-flat contact and cylindrical-on-flat contacts, i.e., fretting-contact-induced crack openings. The crack opening stress of specimen with flat-on-flat contact was lower than those of specimens with cylindrical-on-flat contacts, while that of specimen with 60-mm radius contact pad was lower than that of specimen with 15-mm radius contact pad. The fretting fatigue lives were estimated by integrating the fatigue crack growth curve from an initial propagating crack length to a critical crack length. The predictions of fretting fatigue life with consideration of crack opening were in good agreement with the experimental results.

## 1. Introduction

Fretting is a damage process that occurs at the contact between two components under minute relative motion, while fatigue is a progressive damage that occurs when a component is subjected to cyclic loading. Thus, fretting fatigue is a fatigue failure enhanced by fretting at the contact of components, e.g., bearing and shaft interfaces, bolted and riveted connections, steel cables, and gas turbines [1,2,3]. Fretting fatigue is influenced by cyclic loading (axial, bending, etc.) and contact (contact pressure, geometry, etc.). For flat-on-flat contact, a fretting fatigue crack is likely to nucleate at the location of maximum shear stress range. After crack nucleation, it propagates in the direction of maximum tangential stress range. The influence of tangential stress range decreases as the crack propagates away from the contact. Eventually, the propagating crack is under the domination of bulk alternating stress, and the crack propagates in the direction perpendicular to the bulk alternating stress [4,5,6,7]. Under the combination between contact stress and bulk alternating stress, a fretting fatigue crack nucleates at early stage of fretting fatigue life (*N*_f_). Thus, the fretting fatigue strength is significantly lower than that of plain fatigue. By assuming that *N*_f_ is equal to the fretting fatigue crack propagation life, the fretting fatigue life can be predicted using the linear-elastic fracture mechanics (LEFM) approach [4,5,6,7,8,9,10,11,12].

For components with cylindrical-on-flat contact (bearings, gears, etc.), the Hertzian contact stress is in a function of the normal load, radius of curvature of cylindrical body and modulus of elasticity [13]. It is likely that the Hertzian contact stress can influence the crack nucleation, crack propagation and fretting fatigue life. Hojjati-Talemi et al. [14] investigated the influences of contact geometries (cylindrical-on-flat and flat-on-flat) and pad widths on fretting fatigue behavior of 7075-T6 aluminum alloy. The fretting fatigue crack initiated near the contact trailing edge for all contact geometries. A linear-elastic fracture mechanics approach was applied to model the crack propagation life. For both flat and cylindrical cases, the crack propagation life increased with increased pad width. The difference between the estimated fretting fatigue lives and experimental fretting fatigue lives were about 18%. Hills and Urriolagoitia-Sosa [15] investigated the variables that influence the behavior of a Hertzian type fretting fatigue test. They found that the normal contact load controls the interior stress field and the maximum slip displacement. Araujo et al. [16] applied the Taylor’s point method, i.e., a prediction method for fatigue limit of notched component, in conjunction with the Modified Wohler curve method to predict the high-cycle fatigue strength of cylindrical contacts under a partial slip regime. Their method correctly predicted the failures of Ti-6Al-4V in the medium-cycle fatigue regime, while those in the high-cycle fatigue regime were within an error interval of about ±20%.

Although there are research works related to fretting fatigue [4,5,6,7,8,9,10,11,12,14,15,16,17,18,19,20,21,22,23,24,25], the understanding regarding the crack nucleation, crack path and fatigue life are far from complete. In the present work, the crack nucleation, crack path and fatigue life of cylindrical-on-flat contact fretting fatigue of medium-carbon steel were studied. Bridge-type fretting fatigue experiments were performed using various radii of contact pad. The location of crack nucleation was predicted using finite element analysis (FEA) with the maximum shear stress range criterion and the maximum relative slip amplitude criterion. Crack path was predicted using FEA with the maximum tangential stress (MTS) range criterion. Fretting fatigue life (*N*_f_) was predicted by combining the fatigue crack growth curve (Δ*K*_eff_–da/dN) and the effective stress intensity factor range (Δ*K*_eff_) evaluated by FEA. As a driving force of fatigue crack growth [26], Δ*K*_eff_ was defined as *K*_max_–*K*_op_, where *K*_max_ is the maximum stress intensity factor and *K*_op_ is the stress intensity factor at the crack opening load (*P*_op_). The predictions of crack nucleation, crack path and fatigue life were then compared with those from fretting fatigue experiments. The influence of radius of contact pad on fretting fatigue behavior was then discussed.

## 2. Materials and Methods

### 2.1. Materials

Materials were the medium-carbon steel (JIS S45C steel), i.e., a rod (12-mm diameter) for fretting fatigue specimens and a plate (12-mm thickness) for contact pads. To minimize the influence of residue stress, S45C steel was annealed at 550 °C for 90 min. Tensile tests were performed following the ASTM recommendation [27]. Tensile properties of the annealed plate and rod were almost identical, i.e., σ_Y_ = 478 MPa, σ_U_ = 736 MPa, *E* = 210 GPa and ν = 0.3.

Load-controlled fatigue tests were performed following the ASTM recommendation [28]. Fatigue behavior could be represented by Basquin relationship,

(1) $${\;\sigma }_{a}={\;\sigma \;\prime }_{f}{\left(N_{f}\right)}^{n}$$

where σ_a_ is the stress amplitude, σ′_f_ is the fatigue strength coefficient (681 MPa), and n is the fatigue strength exponent (−0.068). If a specimen does not fail by 2 × 10^6^ cycles, it is considered as a run-out. As a stress amplitude at run-out, the fatigue limit (σ_e_) is 250 MPa.

Fatigue crack growth (FCG) test were performed following the ASTM recommendation [29]. The FCG curve during stable crack growth could be represented by Paris law,

(2) $$\;\frac{da}{dN}=C{\left(\Delta K_{eff}\right)}^{m}$$

where *da*/*dN* is the FCG rate, *C* is the FCG coefficient (3 × 10^−12^ m/cycle), and m is the FCG exponent (3.65). As an effective stress intensity factor range (Δ*K*_eff_) when the crack growth rate is lower than 10^−10^ m/cycle, the threshold effective stress intensity factor range (Δ*K*_eff,th_) is 4 MPa·m^1/2^.

### 2.2. Fretting Fatigue Experiment

**Figure 1:**
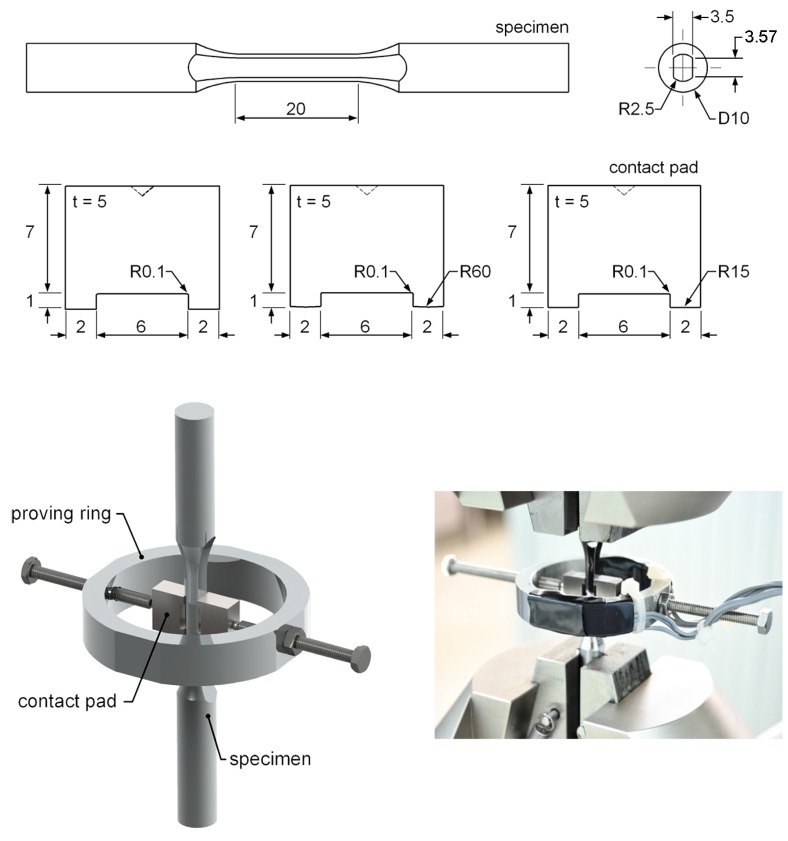
Fretting fatigue experiment (dimensions in mm).

Fretting fatigue experiments with bridge-type contact pads were performed in accordance with the JSME standard [30]. The dimensions of fretting fatigue specimen and contact pad are shown in Figure 1. The thickness of contact pad and the contact length (*l*) were 5 mm and 3.57 mm, respectively. The radius of contact pad (*R*_pad_) was infinity (∞) for the flat-on-flat contact fretting fatigue, while *R*_pad_ were 60 and 15 mm for the cylindrical-on-flat contact fretting fatigues. For flat-on-flat contact (*R*_pad_ = ∞), the perfectly sharp edge is impossible to manufacture, thus the external edge was carefully machined to obtain an edge radius < 20 μm. The set-up of fretting fatigue experiment is also shown in Figure 1. A pair of contact pads was clamped to the gauge part of specimen using clamping screws and a proving ring. Ball joints were used at the interface between the clamping screw and contact pad. The edges of contact pads were placed in the direction perpendicular to the longitudinal axis of specimen. Clamping axis, which passes through the clamping screws, was placed at the middle of specimen. To maintain the alignment between contact pads, clamping screws and proving ring, the contact pads and specimen were carefully machined and assembled using a special fixture.

Full-bridge strain gauges were applied to measure the deformation of proving ring, and then converted to the clamping force. Clamping force could be adjusted using clamping screws. Mean contact pressure (*P*_mean_) is a fraction between the clamping force and cross-sectioned area of pad legs. In the present work, a clamping force (*F*) of 856.8 N, i.e., a mean contact pressure (*P*_mean_) of 60 MPa, was applied using a proving ring. Hertzian contact width (2*b*) and the maximum contact pressure (*P*_max_) could be estimated as follows [31].

(3) $$2b=2{\left[\frac{4R_{pad}}{\pi l}\left(\frac{F}{2}\right)\left(\frac{2\left(1-v^{2}\right)}{E}\right)\right]}^{\frac{1}{2}}$$

(4) $$P_{\max}=\frac{2}{\pi bl}\left(\frac{F}{2}\right)$$

The Hertzian contact widths (2*b*) were 564 and 282 μm for fretting fatigues with *R*_pad_ = 60 and 15 mm, respectively, while the maximum contact pressures (*P*_max_) were 271 and 542 MPa for fretting fatigues with *R*_pad_ = 60 and 15 mm, respectively.

Bulk alternating stresses (σ_B_) with stress amplitudes (σ_a_) of 180 to 300 MPa, frequency of 20 Hz and stress ratio (R) of −1 were applied to the specimens using a fatigue testing machine (Instron: ElectroPuls E10000 with 10-kN load cell, ITW Test & Measurement, Glenview, IL, USA). The temperature and relative humidity during fretting fatigue experiment were controlled at 25 ± 2 °C and 60% ± 5%, respectively. Fatigue life (*N*_f_) is the number of cycles that occur before complete fracture. However, if a specimen does not fail by 2 × 10^6^ cycles, it is considered as a run-out. During fretting fatigue experiments, some specimens were interrupted, and observed under a scanning electron microscope to evaluate the size of contact and the location of crack nucleation. Some specimens were interrupted just before complete fracture, sectioned along the longitudinal axis, and observed under an optical microscope to evaluate the crack path.

## 3. Results and Discussion

### 3.1. Fretting Fatigue Life and Fretting Fatigue Crack

**Figure 2:**
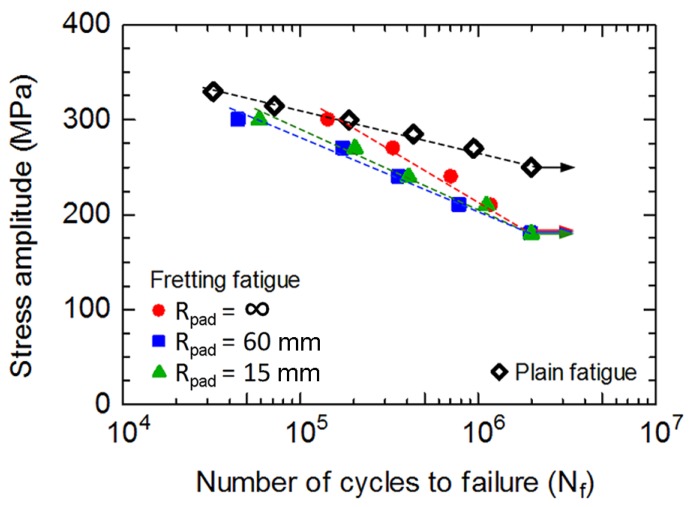
Stress amplitude versus fatigue life.

Stress amplitude (σ_a_) versus fatigue life (*N*_f_) curve is shown in Figure 2. Fatigue life increased with decreasing stress amplitude, which is a typical S-N curve. Fretting fatigue resistances were lower than that of plain fatigue. The specimens with *R*_pad_ = ∞ (flat-on-flat contact) had the highest fatigue resistance following by those with *R*_pad_ = 15 and 60 mm (cylindrical-on-flat contact), respectively. Although similar mean contact pressure was applied for fretting fatigue experiments with various radii of contact pad, the contacts were smaller for the specimens with cylindrical contact pads compared to that with flat contact pad. Thus, the contact pressures at cylindrical-on-flat contacts were higher than that at flat-on-flat contact. High contact pressures of specimens with cylindrical contact pads are the reason for shorter fretting fatigue lives. However, the fatigue limits of fretting fatigue experiments with various radii of contact pad were similar, which is approximately 180 MPa. With the supports for numerical calculations, the influence of contact stress fields with various geometries of contact pad on the crack nucleation and propagation was discussed in the later section.

**Figure 3:**
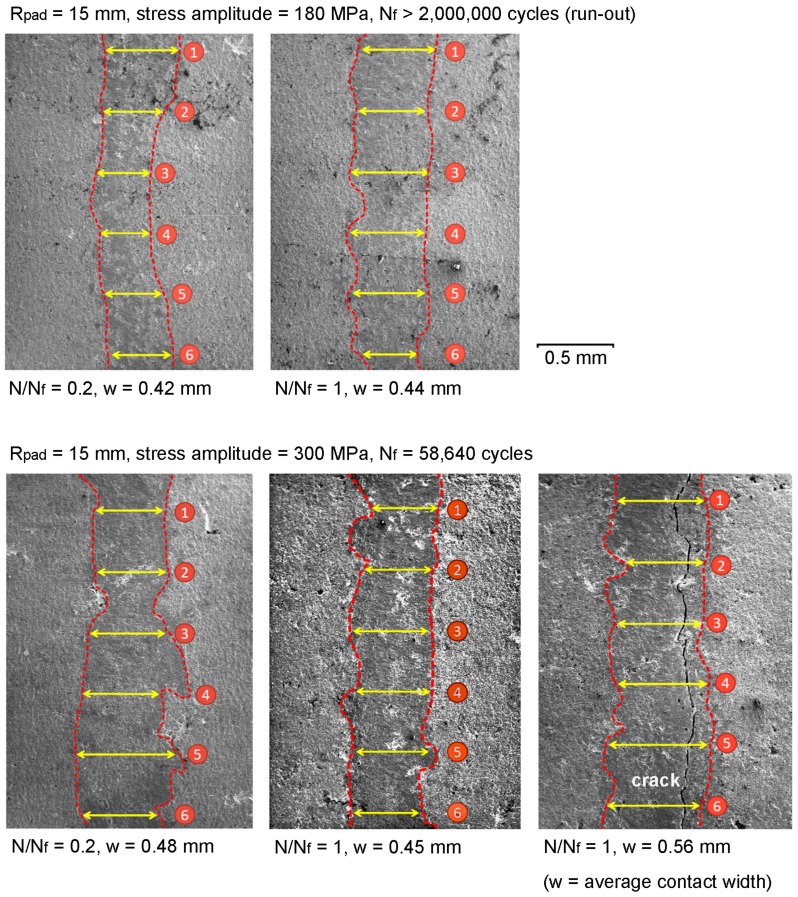
Contact marks on fretting fatigue specimens (*R*_pad_ = 15 mm) tested at stress amplitudes of 180 MPa and 300 MPa (bulk alternating stress is in horizontal direction).

Satoh et al. [32] investigated the influence of contact pad geometry (flat contact, *R* = 2, 4, 8 and 20 mm cylindrical contacts) and mean contact pressure (30 MPa to 1.5 GPa) on fretting fatigue behavior. The contact pad was 12Cr-Mo-W-V steel, while fretting fatigue specimen was 11Cr-Mo-V-Nb steel. They found that the cylindrical contact was plastically deformed due to contact load, and also worn with repeated fretting cycles to become a flat contact. In the present work, some specimens were interrupted, and observed under a scanning electron microscope to evaluate the contacts. The contact marks on fretting fatigue specimens (*R*_pad_ = 15 mm) tested at low and high stress amplitudes (180 MPa and 300 MPa) were observed at *N*/*N*_f_ = 0.2 and 1, as shown in Figure 3. The contact widths at six locations were measured using image analysis software (DinoCapture 2.0, AnMo Electronics Corporation, New Taipei City, Taiwan), and the average contact width (*w*) was determined. Because the high stress amplitude causes large deformation of specimen and large slip amplitude, the contact width of specimen with stress amplitudes of 300 MPa was larger than that of specimen with stress amplitudes of 180 MPa. At same stress amplitude, the contacts without crack at *N*_f_ = 0.2 and *N*_f_ = 1 had similar width. On the other hand, because of the crack opening of specimen tested at stress amplitudes of 300 MPa, the contact with crack was slightly larger than the contact without crack. Because the contact wear was not severe enough to transform the cylindrical-on-flat contact into flat-on-flat contact, the assumption of Hertzian contact stress was applied for the present cylindrical-on-flat contacts.

**Figure 4:**
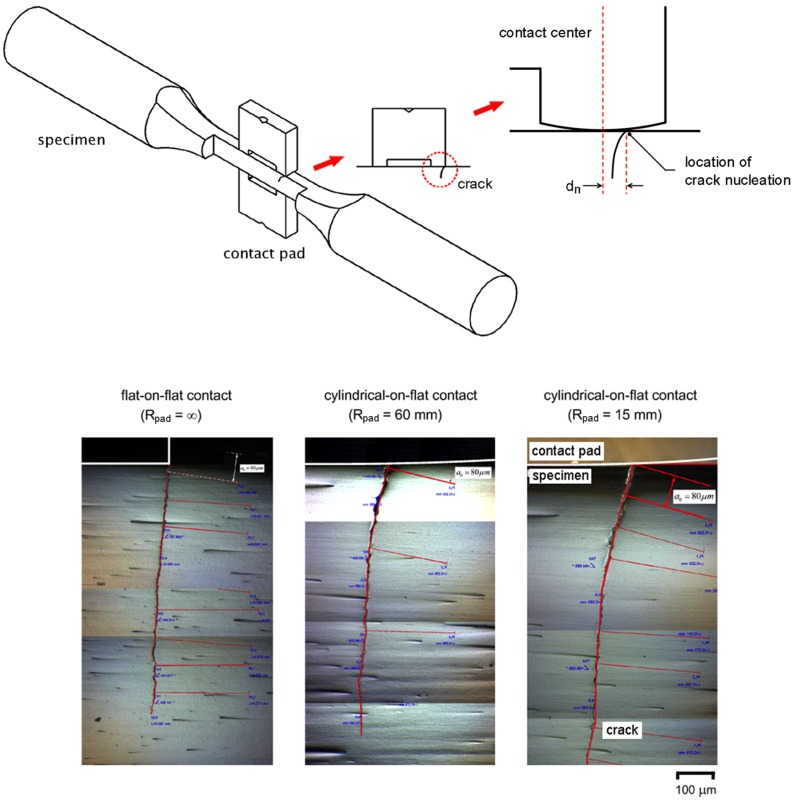
Fretting fatigue cracks tested at a stress amplitude of 300 MPa.

Fretting fatigue experiments at stress amplitude of 300 MPa were interrupted just before complete fracture (*N*/*N*_f_ > 0.9), sectioned along the longitudinal axis using an electrical discharge machine (EDM), and observed under an optical microscope. Micrographs of the longitudinal sections of specimens with *R*_pad_ = ∞, 60 and 15 mm are shown in Figure 4. All fretting fatigue cracks were found to nucleate near the contact trailing edge and propagate at the inclined angle beneath the contact pad. As a reference for the location of crack nucleation, the contact centers of four pad legs were marked on the surface of specimen before fretting fatigue experiment. Distance (*d*_n_) from the contact center to the crack nucleation was measured using image analysis software (DinoCapture 2.0). At stress amplitude of 300 MPa, *d*_n_ were approximately 1000, 210 and 180 μm for specimens with *R*_pad_ = ∞, 60 and 15 mm, respectively.

Nucleation of fretting fatigue crack involves contact stress as well as slip displacement [33]. After crack nucleation, the influence of contact stress on the stress intensity factor at crack tip decreased with crack extension, and then the fretting fatigue crack path gradually changed to the direction normal to the applied bulk alternating stress [4,5,6,7]. To understand the influence of contact geometries on fretting fatigue resistance, the numerical analysis of crack nucleation, crack path, and crack propagation were performed and described in the following sections.

### 3.2. Fretting Fatigue Crack Nucleation

**Figure 5:**
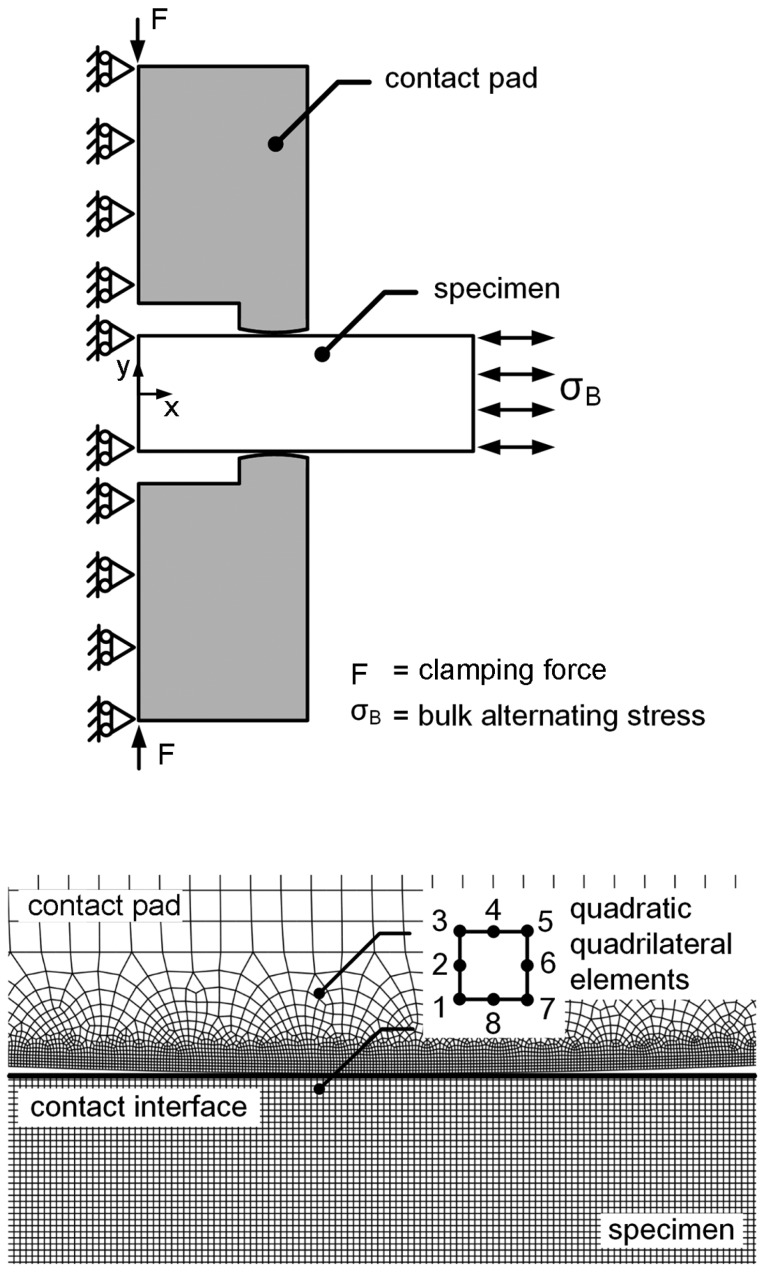
FEA model for the determination of location of crack nucleation.

To evaluate the location of crack nucleation, the model of fretting fatigue experiment without crack was used. Plane-strain linear-elastic FEA, i.e., ABAQUS [34], was applied to determine the stresses and strains around contact. Symmetry model was applied to reduce the calculation time, i.e., a half of the fretting fatigue specimen was used for FEA (Figure 5). Boundary conditions were applied at the middle of fretting fatigue specimen and contact pads, i.e., the displacement in *x* direction and the rotations around *y*-axis were constrained. Frictional-contact elements (quadratic-quadrilateral elements), master-slave algorithm, and penalty method [34] were applied to the contact. The slip of contact is assumed to occur when the shear stress is greater than the critical shear stress (τ_c_), i.e.,

(5) $$\tau \geq \tau_{c}={\mu \sigma }_{n}$$

where σ_n_ and μ are the normal stress and the friction coefficient, respectively. Friction coefficient was measured as μ = 0.6 via a completely sliding wear test [35].

**Figure 6:**
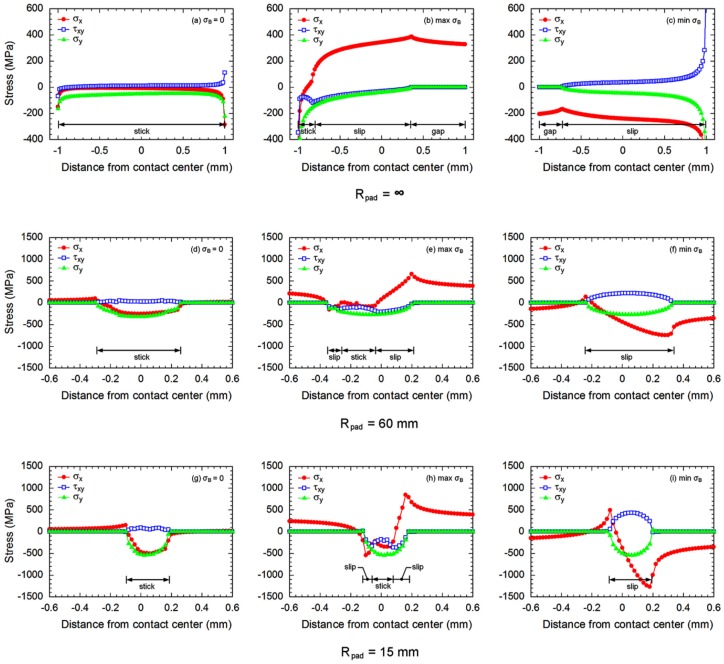
Stresses along contacts of fretting fatigue experiments at a stress amplitude of 300 MPa: (**a**–**c**) *R*_pad_ = ∞, (**d**–**f**) *R*_pad_ = 60 mm, and (**g**–**i**) *R*_pad_ = 15 mm.

Initially, a mean contact pressure (*P*) of 60 MPa was applied to the contact pad. Then, the bulk stress was gradually increased from zero to the maximum σ_B_. Subsequently, the bulk stress was gradually decreased to the minimum σ_B_. Finally, the bulk stress was increased to the zero bulk stress to complete one loading cycle. Bulk stress was varied with intervals of 80 steps for one loading cycle. To minimize the influence of element size, the size of elements was adjusted until the variation of the Von Mises stress around the contact was below 5%. The smallest size of element along contact was 5 μm. Stress distributions along contact at zero σ_B_, the maximum σ_B_ (tension) and the minimum σ_B_ (compression) of fretting fatigue experiment at stress amplitude of 300 MPa are shown in Figure 6.

After applying a mean contact pressure (σ_B_ = 0 of Figure 6), the contact is defined as the region where the σ*_y_* is not zero. For flat-on-flat contact, the contact width was similar to the width of contact pad’s leg, i.e., 2 mm. While, the cylindrical-on-flat contact widths were approximately 550 and 290 μm for fretting fatigues with *R*_pad_ = 60 and 15 mm, respectively. These numerical estimations of cylindrical-on-flat contact widths correspond to the theoretical Hertzian contact widths [31], i.e., 564 and 282 μm for fretting fatigues with *R*_pad_ = 60 and 15 mm, respectively. On the other hand, the maximum compressive stresses (σ*_y_*) were observed at contact centers of cylindrical-on-flat contacts, i.e., 300 and 540 MPa for fretting fatigues with *R*_pad_ = 60 and 15 mm, respectively. These numerical estimations correspond to the theoretical maximum contact pressures [31], i.e., 271 and 542 MPa for fretting fatigues with *R*_pad_ = 60 and 15 mm, respectively.

After applying bulk alternating stress (σ_B_), three types of deformation were observed at contact, i.e., gapping, slipping and sticking. Existences of gapping, slipping and sticking depend on bulk alternating stress, contact pressure, geometries of contact, as well as frictional coefficient. Gapping is defined as the situation when the normal stress perpendicular to contact (σ*_y_*) and shear stress at contact (τ*_xy_*) become zero. In the region where the contact between pad and specimen occurs but the friction is not enough to stick the pad onto specimen, the slipping exists. On the other hand, the sticking between pad and specimen is possible in the region where the friction becomes high enough. Accordingly, the gapping, slipping and sticking could be identified by considering the stress behavior at contact, as shown in Figure 6.

**Figure 7:**
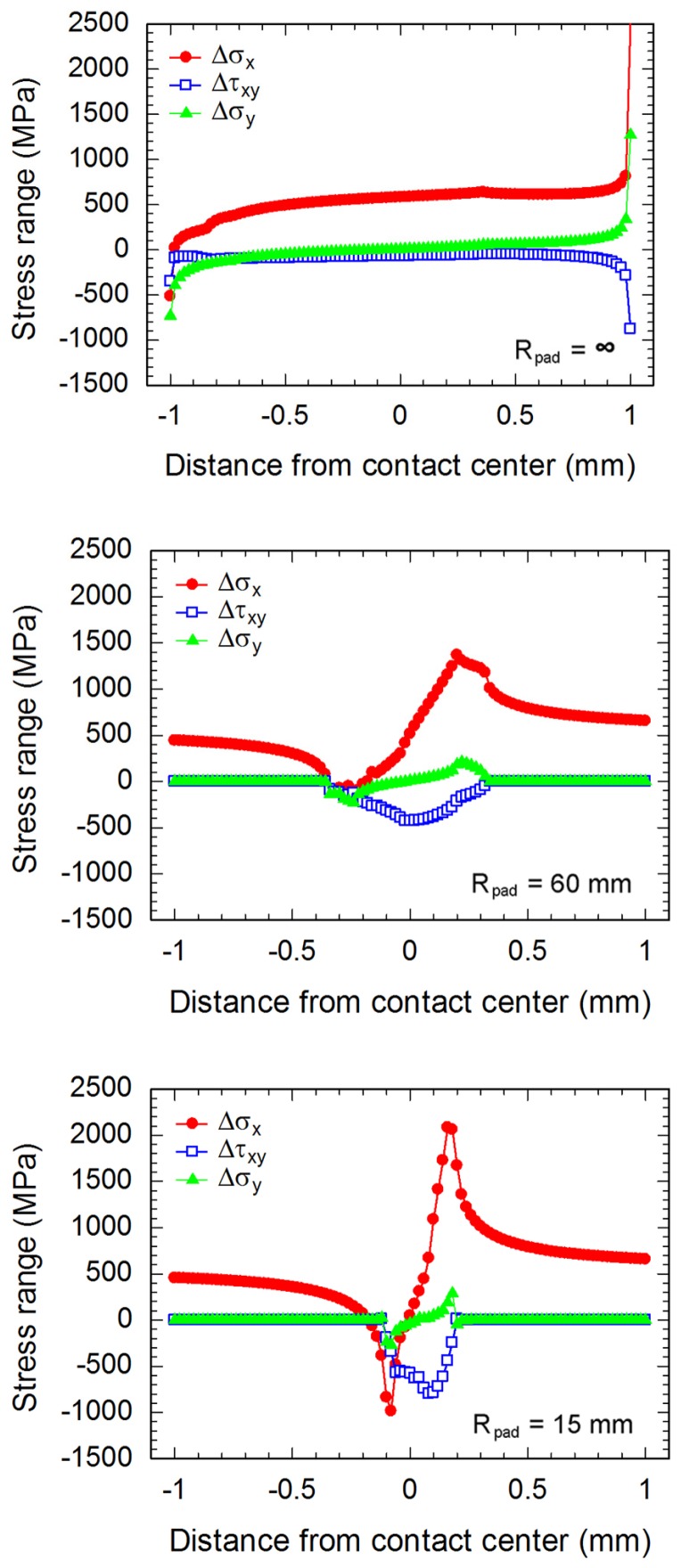
Stress ranges along contacts of fretting fatigue experiments at a stress amplitude of 300 MPa.

As the differences between stresses at the maximum σ_B_ and those at the minimum σ_B_, the distributions of stress range along contacts of fretting fatigue experiments at a stress amplitude of 300 MPa are shown in Figure 7. Contact can be defined as the region where the Δ*σ_y_* is not zero. As an example, the contact width on fretting fatigue specimen (*R*_pad_ = 15 mm) tested at stress amplitude of 300 MPa is 350 μm. However, this contact width is about 22% shorter than that of experiment (Figure 3), i.e., an average contact width of 450 μm. Difference may relate to the plastic deformation of asperities under contact, which was not considered for the present linear-elastic FEA.

**Figure 8:**
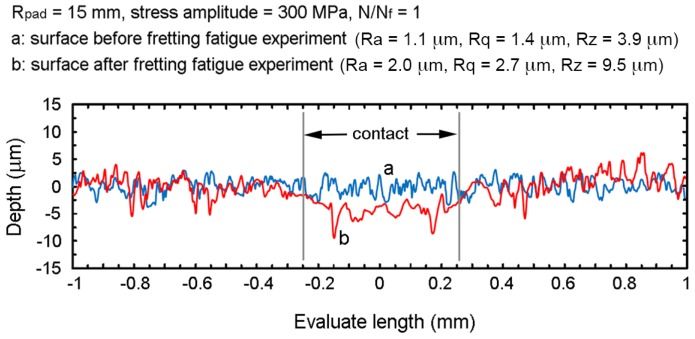
Surface roughness of specimen before and after fretting fatigue experiment (*R*_pad_ = 15 mm and stress amplitude of 300 MPa).

To investigate the influence of surface asperities on contact, the surface roughness of specimen before and after fretting fatigue experiment (*R*_pad_ = 15 mm and stress amplitude = 300 MPa) were measured using a surface roughness tester (Mitutoyo: SJ-310), as shown in Figure 8. The asperities around the contact center were compressed and plastically deformed during fretting fatigue experiment. Moreover, the grooves were observed near the contact trailing edges. Without the consideration of asperities, the contact width from linear-elastic FEA is shorter than that of experiment with deformed asperities. However, it is believed that the stress distributions along contacts without asperities and contact with deformed asperities are marginally different, especially at the early stage of fretting fatigue life. Therefore, the assumption of specimen without asperities was used for the present FEA of crack nucleation.

#### 3.2.1. Maximum Shear Stress Range Criterion

**Figure 9:**
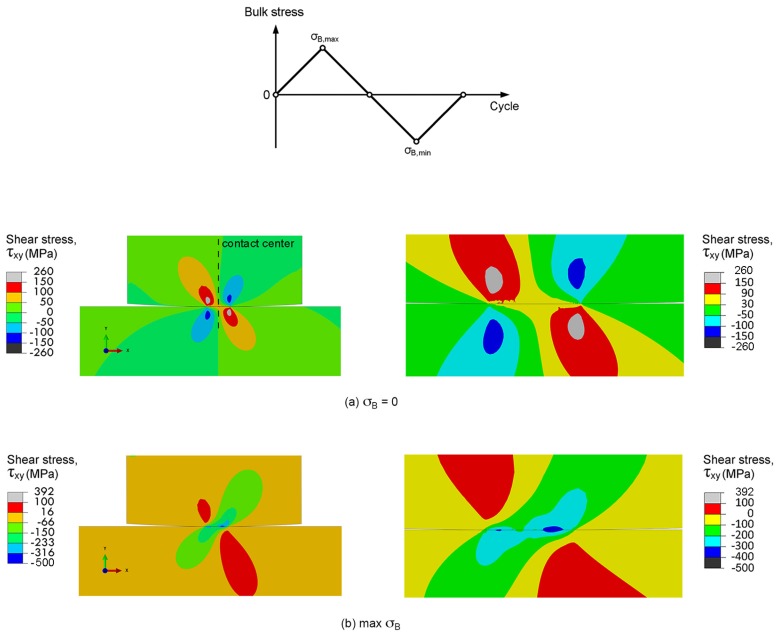

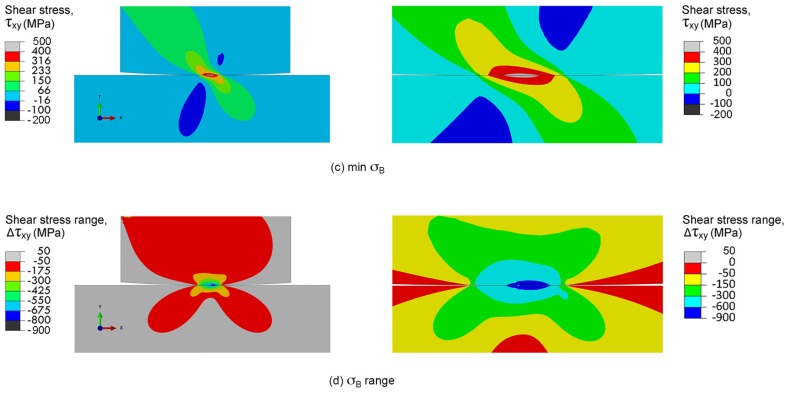
Distributions of shear stresses of fretting fatigue experiment (*R*_pad_ = 15 mm and σ_a_ = 300 MPa): (**a**) σ_B_ = 0; (**b**) the maximum σ_B_; (**c**) the minimum σ_B_; and (**d**) shear stress ranges.

It is known that the cycle stress causes the irreversibility of shear displacement at the surface of alloy, i.e., intrusions and extrusions. The stress concentration at these sites can promote fatigue crack nucleation [36]. Thus, the fretting fatigue crack is assumed to nucleate at the location of the maximum shear stress range, i.e., the maximum shear stress range criterion. The shear stress range at contact was evaluated using plane-strain linear-elastic FEA. The distributions of shear stresses around contact of right leg of contact pad during fretting fatigue experiment (*R*_pad_ = 15 mm and *σ*_B_ = 300 MPa) are shown in Figure 9. After applying of a contact pressure (*σ*_B_ = 0), the highest shear stress occurred below the contact (Figure 9a), which is the typical Hertzian contact stress for cylindrical-on-flat contact. However, the highest shear stress moved to the contact with the contributions of bulk alternating stress and friction at contact. The highest shear stress at the maximum *σ*_B_ was observed on the contact and at the right side of contact center (Figure 9b), while that at minimum *σ*_B_ was observed on the contact and at the left side of contact center (Figure 9c). As the difference between shear stresses at the maximum and minimum σ_B_, the distribution of shear stress ranges is shown in Figure 9d. The maximum shear stress range was observed on the contact and at the right side of contact center, i.e., near the contact trailing edge. The distributions of shear stresses around contacts of fretting fatigue specimens (*σ*_B_ = 300 MPa) with various radii of contact pads were numerically calculated. Distances from the contact center to the location of the maximum shear stress (*d*_n_) were approximately 1000, 20 and 80 μm for specimens with *R*_pad_ = ∞, 60 and 15 mm, respectively.

#### 3.2.2. Maximum Relative Slip Amplitude Criterion

**Figure 10:**
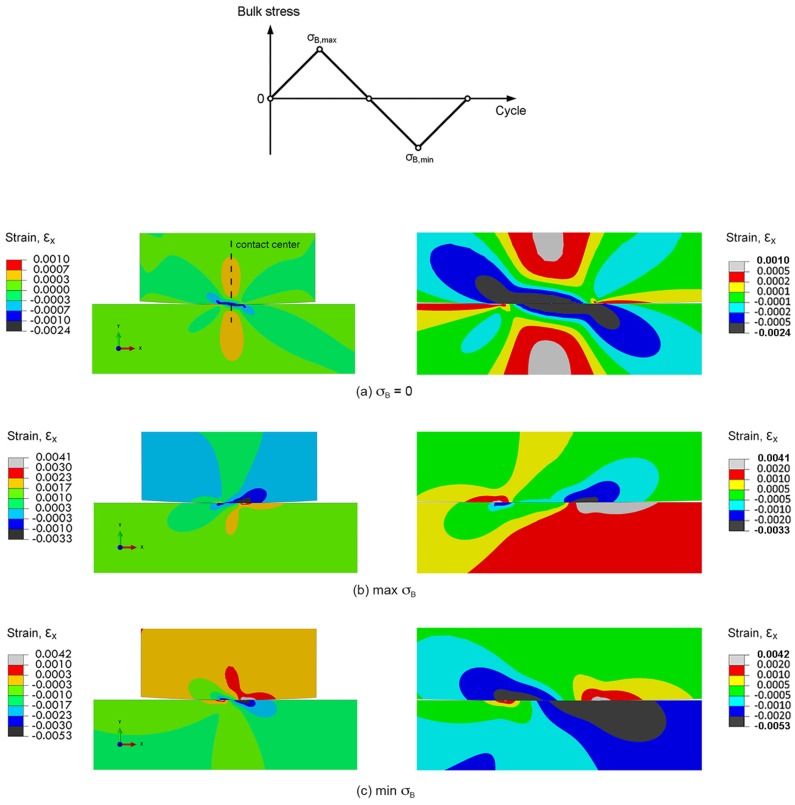
Distributions of normal strains in *x*-direction of fretting fatigue experiment (*R*_pad_ = 15 mm and σ_a_ = 300 MPa): (**a**) σ_B_ = 0; (**b**) the maximum σ_B_; and (**c**) the minimum σ_B_.

Slipping at contact can cause contact wear, which enhance the stress concentration at wear track. The stress concentration at these sites may promote fatigue crack nucleation. Thus, the fretting fatigue crack is assumed to nucleate at the location of the maximum relative slip amplitude, i.e., the maximum relative slip amplitude criterion. The slipping of contact was evaluated using plane-strain linear-elastic FEA. To understand the slipping of contact, the distributions of normal strains in *x*-direction (ε*_x_*) during fretting fatigue experiment (*R*_pad_ = 15 mm and σ_a_ = 300 MPa) are shown in Figure 10. After applying a contact pressure (σ_B_ = 0), the distributions of ε*_x_* along contact of contact pad and specimen were similar (Figure 10a), i.e., sticking. At the maximum σ_B_, the highest ε*_x_* along contact of specimen was observed at the right side of contact center (Figure 10b), while that at minimum σ_B_ was observed at the left side of contact center (Figure 10c). The mismatches in ε*_x_* between contact pad and specimen, i.e., slipping, were observed along contacts (Figure 10b,c), especially at the outer regions of contacts.

**Figure 11:**
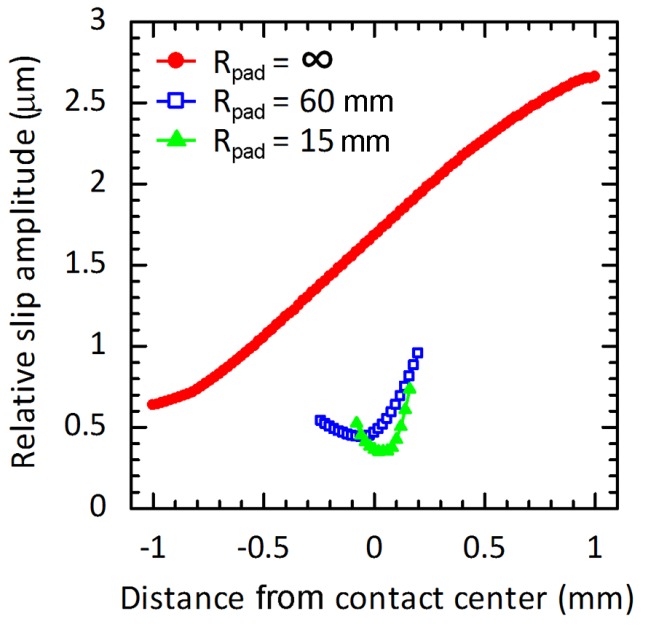
Relative slip amplitudes along contacts of fretting fatigue experiments at a stress amplitude of 300 MPa.

As a difference in deformations in *x*-direction between the contact points on contact pad and on specimen, the relative slips at the maximum and minimum σ_B_ were obtained. Subsequently, the relative slip range, i.e., a total slip from the minimum to the maximum of σ_B_, and the relative slip amplitude, i.e., a half of relative slip range, could be determined. To verify the FEA of relative slip amplitude, the relative slip amplitude at external contact edge of flat-on-flat contact (*R*_pad_ = ∞) was measured using an extensometer, and compared with that from FEA. Both the FEA and the experimental results were in good agreement [6]. Under stress amplitude of 300 MPa, the relative slip amplitudes along various contacts are shown in Figure 11. The influence of radius of contact pad on the relative slip amplitudes could be clearly observed in the figure. Distances from the contact center to the location of the maximum relative slip amplitude (*d*_n_) were approximately 1000, 200 and 160 μm for specimens with *R*_pad_ = ∞, 60 and 15 mm, respectively.

**Figure 12:**
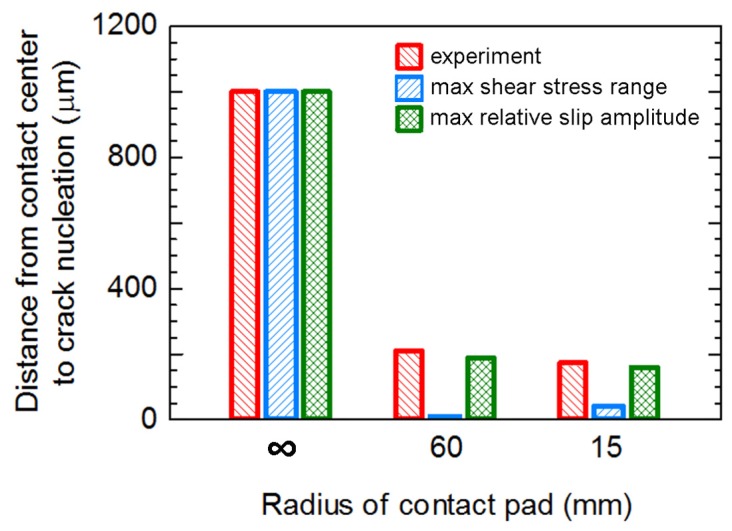
Distances from the contact center to the crack nucleation of fretting fatigue experiments at a stress amplitude of 300 MPa.

Distances from the contact center to the location of crack nucleation (*d*_n_) obtained from experiments and FEAs, i.e., the maximum shear stress range criterion and the maximum relative slip amplitude criterion, were compared, as shown in Figure 12. The locations of crack nucleation of flat-on-flat contact (*R*_pad_ = ∞) using the maximum shear stress range criterion and the maximum relative slip amplitude criterion were similar and correspond to that of experiment, i.e., crack nucleated at the external contact edge. On the other hand, the locations of crack nucleation of cylindrical-on-flat contacts (*R*_pad_ = 60 and 15 mm) using the maximum shear stress range criterion and the maximum relative slip amplitude criterion were different. The predicted locations of crack nucleation using the maximum relative slip amplitude criterion were in better agreement with the experimental results. Therefore, the maximum relative slip amplitude criterion should be used for the prediction of the location of fretting fatigue crack nucleation under cylindrical-on-flat contact.

### 3.3. Fretting Fatigue Crack Path

The effect that the model’s geometry has on fretting fatigue life predictions was studied by Vazquez et al. [37], where the crack initiation and crack-propagation phases were combined to obtain the total fatigue life. Their results showed solid predictions for both 2D and 3D FEM models when considering a semi-elliptical surface crack. The use of a 2D FEM model, while incorporating a semi-elliptical surface crack, is therefore justified because it provides the advantage of lower computational cost compared with 3D FEM model. In the present study, the fretting fatigue crack could be observed in the early stage of fatigue life; the crack nucleation life was significantly short and the fretting fatigue life was dominated by the crack propagation [1,7,38]. Accordingly, the 2D FEA model of fretting fatigue experiment with through-thickness crack was used for the evaluation of crack path and crack propagation.

**Figure 13:**
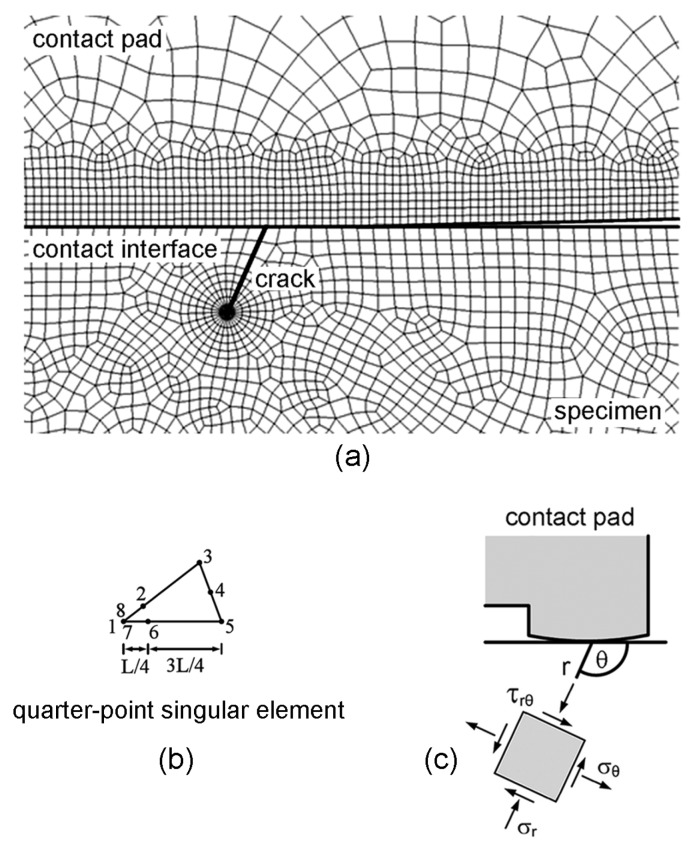
(**a**) FEA model for the determination of fatigue crack path; (**b**) element type at crack tip; and (**c**) polar coordinate at contact.

As an initial propagating crack, a crack in the direction of the maximum tangential stress range (Δσ_θmax_) was introduced at the location of the maximum relative slip amplitude. Subsequently, it is assumed to propagate in the direction of Δσ_θmax_, i.e., the maximum tangential stress range criterion [39]. Stresses at crack tip were evaluated using plane-strain linear-elastic FEA (Figure 13a). To reduce the calculation process time, a half of the fretting fatigue specimen with a crack was used for FEA. Quarter-point singular elements were applied at crack tip (Figure 13b), while the quadratic quadrilateral elements were used for the remaining elements. Polar coordinates were applied to numerically evaluate the inclined angle (θ) of an initial propagating crack, as shown in Figure 13c.

According to the fracture mechanics approach for a small fretting fatigue crack [4,40], the length of an initial propagating crack could be assumed as the critical smallest crack length (*a*_o_), i.e.,

(6) $$a_{o}=\frac{1}{\pi }{\left(\frac{\Delta K_{th}}{\Delta \sigma_{e}}\right)}^{2}$$

where Δσ_e_ and Δ*K*_th_ are the fatigue limit of plain fatigue and the threshold stress intensity factor range, respectively. For JIS S45C steel, Δσ_e_ and Δ*K*_th_ were 250 MPa and 4 MPa·m^1/2^, respectively. Thus, *a*_o_ was given as 80 μm. In the previous study [6,41], the sensitivities of path radius and length of propagating crack on fretting fatigue crack path were evaluated. The FEA result corresponded to the experimental result when *a*_o_/2 was used as: (i) the path radius for calculating the crack orientation; and (ii) the length of propagating crack for every FEA step. Accordingly, a similar procedure of crack path prediction was applied here.

**Figure 14:**
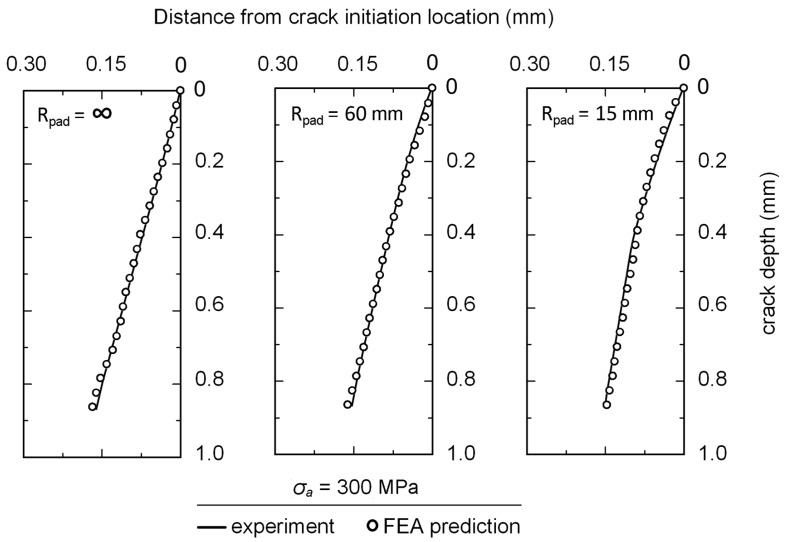
Fretting fatigue crack paths of fretting fatigue experiments at a stress amplitude of 300 MPa.

Size of elements was adjusted until the variation of stress intensity factor at crack tip was lower than 5%, i.e., the smallest size of element at crack tip was 1 μm. Based on path radius of *a*_o_/2, the direction of Δσ_θmax_ was determined and the crack was extended by *a*_o_/2. This process was repeated for each FEA step to obtain a fretting fatigue crack path. Comparisons between the predicted crack paths and the experimental crack paths at a stress amplitude of 300 MPa are shown in Figure 14. Predicted crack paths were in good agreement with the experimental results.

### 3.4. Fretting-Contact-Induced Crack Opening/Closure

It is known that the FCG behavior may be influenced by the crack opening/closure behavior, and the effective stress intensity factor range (Δ*K*_eff_) should be applied as the driving force of FCG [26]. In the previous study [38], the crack opening under compressive bulk alternating stress was found from both FEA and experiment, i.e., an elastic compliance method was applied to measure the crack opening of bridge-type fretting fatigue experiment. The restraint of deformation of contact region side of crack surface, which was induced by the contact pressure and tangential stress, was the dominant cause of the crack opening under compressive bulk alternating stress, i.e., the fretting-contact-induced crack opening/closure.

**Figure 15:**
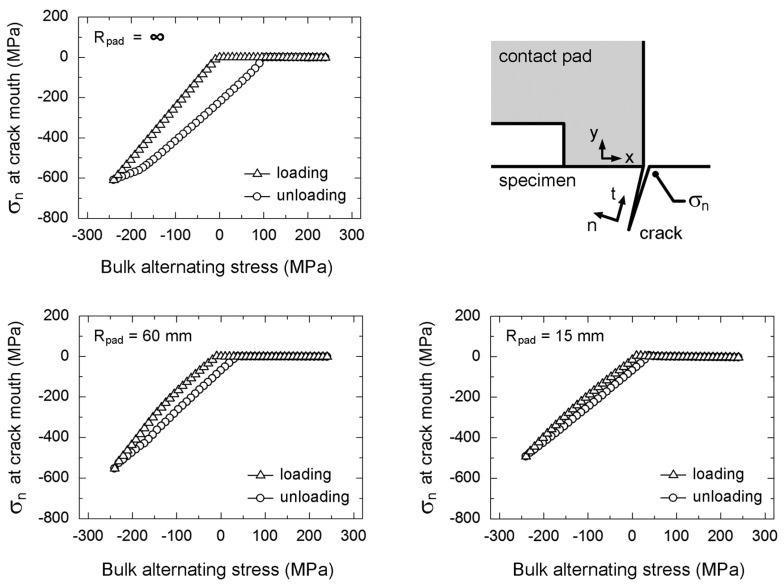
Stresses near the crack mouth of a 80 μm fretting fatigue crack tested at a stress amplitude of 300 MPa.

Accordingly, the influence of geometry of contact pad on the fretting-contact-induced crack opening/closure was evaluated using FEA. Plane-strain linear-elastic FEA was performed using a half model of fretting fatigue specimen with a crack. Frictionless contact is assumed for the contact between crack surfaces. The stresses (σ_n_) at 10 μm away from the crack mouth of fretting fatigue specimen with an 80-μm crack tested at a stress amplitude of 300 MPa are shown in Figure 15. The opening of crack surfaces is the situation when the stress normal to crack surface (σ_n_) at 10 μm away from the crack mouth becomes zero. The crack opening and closing at crack mouth of fretting fatigue specimen occurred at different bulk alternating stresses, i.e., crack closing is possible under a tensile bulk alternating stress, while crack opening is possible under a compressive bulk alternating stress. The opening stress was influenced by the radius of contact pad (*R*_pad_), i.e., the opening stress of specimen with *R*_pad_ = ∞ (flat-on-flat contact) occurred at the lowest compressive bulk alternating stress following by those of specimens with *R*_pad_ = 60 and 15 mm (cylindrical-on-flat contacts), respectively.

In the present study, the effective stress intensity factor range (Δ*K*_θmax,eff_) was used as a driving force for the FCG with the fretting-contact-induced crack opening/closure. The contour integral method [34] was applied for FEA of the maximum tangential stress intensity factor (*K*_θmax_), which can be given as:

(7) $$\sigma_{\theta \max}=\frac{1}{\sqrt{2\pi r}}\cos\frac{\theta }{2}\left(K_{\theta \max}\cos^{2}\frac{\theta }{2}\right)$$

The effective stress intensity factor range (Δ*K*_θmax,eff_) can be determined as:

(8) $$K_{\theta \max,\mathrm{eff}}=K_{\theta \max,\max}-K_{\theta \max,\mathrm{op}}$$

where *K*_θmax,max_ is the maximum tangential stress intensity factor at the maximum bulk alternating stress, and *K*_θmax,op_ is the maximum tangential stress intensity factor at crack opening stress, which can be determined from the change in slope during loading of Figure 15.

**Figure 16:**
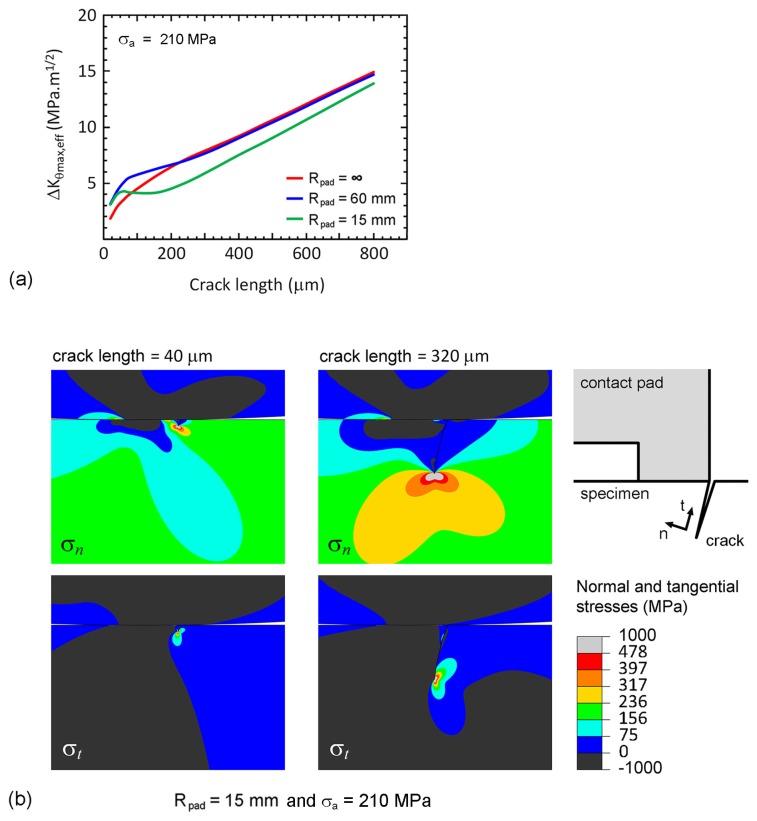
Fretting fatigue at stress amplitude of 210 MPa: (**a**) relationships between the effective stress intensity factor range and crack length; and (**b**) distributions of normal and tangential stresses on fretting fatigue specimen with 15-mm radius of contact pad at the maximum σ_B_.

Relationships between the effective stress intensity factor range (Δ*K*_θmax,eff_) and crack length (*a*) of fretting experiments at stress amplitude of 210 MPa are shown in Figure 16a. At the beginning of crack propagation, i.e., *a* < 100 μm, the crack tips of cylindrical-on-flat contacts were outside the region of Hertzian contact stress, thus the Hertzian contact stress enhanced the crack propagation, i.e., Δ*K*_θmax,eff_ of cylindrical-on-flat contacts were higher than that of flat-on-flat contact. As an example, the distributions of normal and tangential stresses on fretting fatigue specimen (*R*_pad_ = 15 mm) with a 40-μm crack at the maximum σ_B_ are shown in Figure 16b.

As crack propagated longer than 100 μm, the crack tip of cylindrical-on-flat contact was eventually surrounded by the Hertzian contact stress (*a* = 320 μm in Figure 16b). The Hertzian contact stress retarded the crack propagation, and the increasing rate of Δ*K*_θmax,eff_ with crack length decreased. Since the number of cycles during the beginning of crack propagation (a < 100 μm) dominated the fatigue life, the fatigue resistance of flat-on-flat contact (low Δ*K*_θmax,eff_) became higher than those of cylindrical-on-flat contacts (high Δ*K*_θmax,eff_). When comparing between cylindrical-on-flat contacts with *R*_pad_ = 60 and 15 mm, Δ*K*_θmax,eff_ of specimen with *R*_pad_ = 60 mm was higher than that of specimen with *R*_pad_ = 15 mm (Figure 16a). Therefore, the fatigue resistance of cylindrical-on-flat contact with *R*_pad_ = 60 mm was slightly lower than that of cylindrical-on-flat contact with *R*_pad_ = 15 mm. These findings corresponded to the fretting fatigue lives from experiments (Figure 2).

### 3.5. Prediction of Fretting Fatigue Life

**Figure 17:**
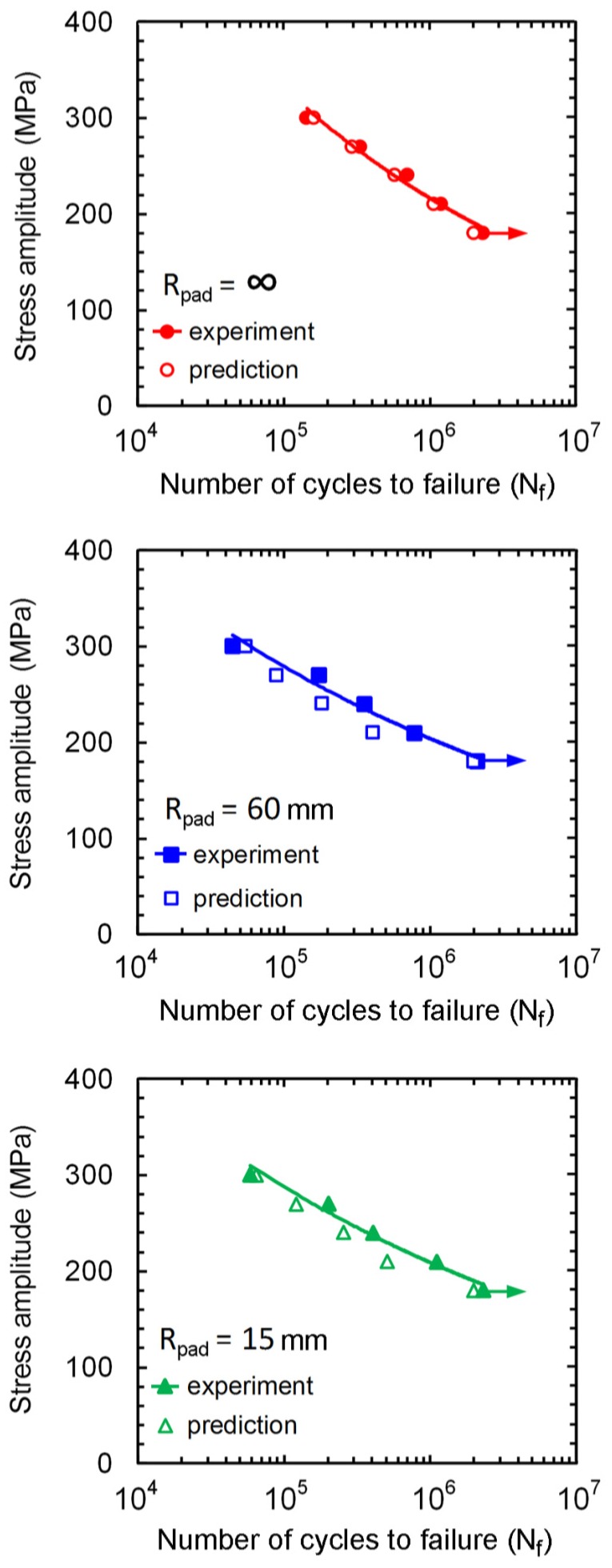
Fretting fatigue lives.

Because the crack nucleation life was significantly short, the fretting fatigue life (*N*_f_) was dominated by the crack propagation. Thus, the crack propagation life from an initial propagating crack length to a critical crack length was assumed to be the *N*_f_,

(9) $$N_{f}=\int_{\frac{a_{o}}{2}}^{a_{c}}\frac{da}{C{\left(\Delta K_{\theta \max,\mathrm{eff}}\right)}^{m}}$$

where *a*_o_/2 is the length of the initial propagating crack, while a_c_ is the critical crack length for fatigue failure, i.e., the crack length at which *K*_θmax,max_ reaches a *K*_IC_ of 15 MPa·m^1/2^. Comparisons between the predicted and experimental *N*_f_ are shown in Figure 17. The *N*_f_ predicted using Δ*K*_θmax,eff_ are in good agreement with the experimental results. However, the predicted fatigue lives are slightly shorter than the experimental fatigue lives. It is believed that the predictions can be improved if the plasticity-induced crack closure at the tip of propagating crack is considered in the numerical predictions.

## 4. Conclusions

Fretting fatigue experiments and finite element analysis (FEA) were carried out to investigate the influence of cylindrical-on-flat and flat-on-flat contacts on fretting fatigue behavior of medium-carbon steel. The location of fretting fatigue crack nucleation was predicted using the maximum shear stress range criterion and the maximum relative slip amplitude criterion. The fretting fatigue crack path was predicted using the maximum tangential stress range criterion. Then, the fretting fatigue lives (*N*_f_) were estimated using the effective stress intensity factor range (Δ*K*_θmax,eff_). The main conclusions are as follows.

1. Fretting fatigue resistance increased with the radius of contact pad (*R*_pad_), i.e., the specimens with *R*_pad_ = ∞ (flat-on-flat contact) had the highest fatigue resistance following by those with *R*_pad_ = 15 and 60 mm (cylindrical-on-flat contacts), respectively.
2. Locations of fretting fatigue crack nucleation of flat-on-flat contact (*R*_pad_ = ∞) predicted by using the maximum shear stress range criterion and the maximum relative slip amplitude criterion were similar, and corresponded to that of experiment. However, the locations of fretting fatigue crack nucleations of specimens with cylindrical-on-flat contacts (*R*_pad_ = 60 and 15 mm) predicted by using the maximum shear stress range criterion and the maximum relative slip amplitude criterion were significantly different. The predictions by using the maximum relative slip amplitude criterion gave the better agreement with the experimental results. Therefore, the maximum relative slip amplitude criterion should be used for the prediction of the location of fretting fatigue crack nucleation under cylindrical-on-flat contact. Based on the maximum tangential stress range criterion, the predicted crack paths were in good agreement with the experimental results.
3. Fretting-contact-induced crack opening under compressive bulk alternating stress could be found from FEA. Opening stress was influenced by the radius of contact pad (*R*_pad_), i.e., the opening stress of specimen with *R*_pad_ = ∞ (flat-on-flat contact) occurred at the lowest compressive bulk alternating stress following by those of specimens with *R*_pad_ = 60 and 15 mm (cylindrical-on-flat contacts), respectively. The predictions of fretting fatigue life (*N*_f_) with consideration of crack opening, i.e., the effective stress intensity factor range (Δ*K*_θmax,eff_), were in good agreement with the experimental results.
